# Bi-Axial Buckling of Laminated Composite Plates Including Cutout and Additional Mass

**DOI:** 10.3390/ma12111750

**Published:** 2019-05-29

**Authors:** Anish, Abhay Chaubey, Ajay Kumar, Bartłomiej Kwiatkowski, Danuta Barnat-Hunek, Marcin K. Widomski

**Affiliations:** 1 Department of Civil Engineering, Birla Institute of Technology Mesra, Patna Campus, Patna 800014, India; anish_85civil@yahoo.co.in; 2 Department of Civil Engineering, Koneru Lakshmaiah Education Foundation, Vaddeswaram 522502, India; abhaychaubey26@gmail.com; 3 Department of Civil Engineering, National Institute of Technology Patna, Patna 800005, India; 4 Faculty of Civil Engineering and Architecture, Lublin University of Technology, Nadbystrzycka St. 40, 20-618 Lublin, Poland; b.kwiatkowski@pollub.pl (B.K.); d.barnat-hunek@pollub.pl (D.B.-H.); 5 Faculty of Environmental Engineering, Lublin University of Technology, Nadbystrzycka St. 40B, 20-618 Lublin, Poland

**Keywords:** buckling, laminated composite plates, cutout, additional mass

## Abstract

In the presented paper, a study of bi-axial buckling of the laminated composite plate with mass variation through the cutout and additional mass is carried out using the improved shear deformation theory (ISDT). The ISDT mathematical model employs a cubic variation of thickness co-ordinates in the displacement field. A realistic parabolic distribution of transverse shear strains through the plate thickness is assumed and the use of shear correction factor is avoided. A C° finite element formulation of the mathematical model is developed to analyze the buckling behavior of laminated composite plate with cutout and additional mass. As no results based on ISDT for the considered problem of bi-axial buckling of the laminated composite plate with mass variation are available in the literature, the obtained results are validated with the data available for a laminated composite plate without cutout and additional mass. Novel results are obtained by varying geometry, boundary conditions and ply orientations.

## 1. Introduction

The composite materials proved to be much more efficient than the traditional materials that led to its wide use as a structural element around the world. Nowadays fiber reinforced polymer composites constitute the dominant materials applied in industry, e.g., the aerospace industry. Composites consist of a combination of two or more materials with different properties. They contain various fibers, which may be metallic or non-metallic, as well as grains of various materials and flakes. Distribution of these components may be homogenous or non-homogeneous. Out of all composite materials, laminated composites are very popular in industries. Excellent properties, such as high strength, high strength to weight ratio and low weight make them more popular in contemporary industrial environments [1]. They are characterized by high stiffness, damping and good directional properties. The fiber in composites is the main load-bearing constituent which is stronger and stiffer than other structural materials. For this reason, composites are used in structural elements, marine parts, aircrafts, etc.

Certain properties, such as static, dynamic and damping are very important [1,2]. In addition, there are notches in these laminated boards, because the cutouts can be used as doors and windows, ports for mechanical and electrical systems or holes for damage control, etc. The advantage of cutting is a reduction in the total mass that significantly affects the reaction to buckling, as well as reduces total stiffness and bending behavior.

Various studies were carried out in the area of laminated composite and hybrid composite materials in the recent years [3,4,5,6]. Bending and twisting characteristics were initially investigated using complex theories. Later, some researchers agreed to develop an improved and simplified theory. Various theories were proposed to analyze laminated composite plates. The simplest theory based on the displacement field is the classical laminated plate theory (CLT). The CLT is based on the Kirchhoff assumptions and neglects the effects of transverse shear stresses and underestimates deflections but overestimates natural frequencies and buckling loads [7].

Ashton and Whitney [8] were the first to analyze laminated composite panels using classical plate theory for plate deformation modeling. Reissner [9,10] presented a constant theory containing the effects of shear strain for the first time. The main assumption made in this study gave a coherent representation of stress distribution over the entire thickness. The same degree of approximation was employed by Mindlin [11] on the kinematic assumptions of the displacement fields given by Reissner [9,10], which resulted in obtaining the governing equations from a direct method for analysis of thin and thick plates considering transverse shear effects. However, maintaining the zero shear force condition at the top and bottom of the laminated plate required the involvement of shear correction factors.

The first order shear strain (FSDT) theory, in which transverse shear stresses are considered, is used to analyze thick plates [12]. The FSDT assumes constant shear stresses through the thickness of the plate [6]. The accuracy of FSDT predictions depends to a large extent on the shear correction factor [12,13]. In order to develop a theory involving shear deformation and rotational inertia, Whitney and Pagano [14] expanded the work of Yang et al. [15]. A high-shear strain (HSDT) theory was proposed [16] to circumvent the limitations of the FSDT method, Reddy [17] developed a simple higher order theory to analyze laminated composite panels. This theory contained the same independent unknown as in the FSDT and considered the parabolic distribution of shear stress through plates thickness with zero transverse shear stresses on the upper and lower surface of the plate without the participation of shear correction coefficients. The HSDT gives a non-linear distribution of transverse shear stresses by the thickness of the plate when the boundary conditions are met. Therefore, there is no need to apply the shear correction factor in the HSDT. The origin of higher shear deformation theories (HSDT) goes back to the work of Hildebrand et al. [18] who made significant contributions by dispensing all the assumptions of Kirchoff’s plate theory. Kant et al. [19] derived the complete set of equations of an isotropic version of the Lo et al. [20] theory and presented extensive numerical results with a proposed numerical integration technique. The general finite element formulation for the plate bending problem based on a higher-order displacement model and a three-dimensional state of stress and strain was devised. The theory incorporates linear and quadratic variations of transverse normal strain and transverse shearing strains and stresses, respectively through the thickness of the plate [19]. Swaminathan and Patil [21] proposed a refined higher-order model to solve the natural frequency of simple supported anti-symmetric sandwich composites and sandwich panels.

Due to an infringement of the continuity of transverse stresses on the border of the contact layers and the involvement of complex models, researchers suggested various forms of the improved theory of a higher order. In order to gain access to the buckling behavior of laminated composites, different models were proposed using different theories of different researchers. Baharlou [22] presented a method of analysis of free vibration and buckling of laminated composite panels. Nguyen-Van et al. [23] conducted the buckling and vibration analysis of laminated composite plate/shell structures via a smoothed quadrilateral flat shell element with in-plane rotations. Other research used the Ritz method to model the simply supported, rectangular, laminated composite plates to calculate the critical buckling loads subjected to different loading conditions [24].

Harris [25] considered the buckling and post-buckling behavior of orthotropic laminated plates. Aydin et al. [26] carried out a numerical buckling analysis of laminated composite plates with an elliptical/circular cutout using a finite element method (FEM). The FEM dominates numerical structural analysis due to extensive research background [6,27]. Other researchers produced an analytical solution under random conditions of axial loading, internal pressure, and in-plane shear loading to study the effects of buckling responses on cylindrical plates [28].

The paper by Zhai et al. [29] deals with the free vibration analysis of two kinds of five-layered composite sandwich plates with two-layered viscoelastic cores based on the first-order shear deformation theory. Vescovini and Dozio [30] presented a unified Lévy-type solution procedure using both layerwise and equivalent single layer theories for the buckling analysis of thin and thick composite plates under biaxial loads. Raju et al. [31] carried out an optimization study for the post-buckling design of orthotropic variable angle tow composite plates under axial compression. Nguyen et al. [32] brought a unified framework on higher order shear deformation theories (HSDTs), modeling and analysis of laminated composite plates. The major objective of their work was to unify all higher order shear deformation theories in a unique formulation by a polynomial form and to propose the new higher shear deformation models systematically based on a unified formulation. In addition, the effect of thickness stretching was taken into account by considering a quasi-3D theory. It was found that the unique formulation of a polynomial form could theoretically cover all existing HSDTs models and was sufficient to describe the nonlinear and parabolic variation of transverse shear stress [32]. Alesadi et al. [33] employed the Isogeometric approach (IGA) and Carrera’s Unified Formulation (CUF) for free vibration and linearized buckling analysis of laminated composite plates. The CUF presents an effective formulation to employ any order of Taylor expansion for the analyses of two-dimensional plate models. Higher-order theories supposed by CUF are free from Poisson locking phenomenon and they do not require any shear correction factor. Therefore, combining IGA and CUF provides a suitable methodology to analyze laminated plates [33]. Various works done by researchers [34,35,36,37,38,39] on FE (Finite Element) modelling of laminated composite plates/shells.

A 3D buckling analysis of thick orthotropic rectangular plates was investigated by Srinivas and Rao [40]. Their linear and small deformation theory of elasticity solution accounted for all the nine elastic constants of orthotropy. For the buckling analysis of elastic plates, Reddy and Pan (Reddy & Phan, Ref. [41] used a higher order shear deformation theory (HSDT). They compared with classical plate theory (CPT), first order shear deformation theory (FSDT) and exact solution. Their theory predicts buckling of the plate more accurately then CPT and FSDT. Khdeir and Librescu [42] also used higher order plate theory to study buckling analysis of asymmetric cross-ply laminated plate considering a variety of boundary conditions. A CPT for buckling analysis of thin skew laminates were presented by Wang [43] based on B-spline Rayleigh-Ritz method. Fares and Zenkour [44] presented various plate theories for buckling analysis of non-homogeneous laminated composite cross-ply plates. The critical buckling loads of laminated skew plates subjected to in-plane compression was presented by Hu and Tzeng [45] using ABAQUS. Chakrabarti and Sheikh [46] used Reddy’s HSDT by implementing a new triangular element to study buckling of composite laminates. Zhen and Wanji [47] computed critical buckling load of composite laminates and sandwich laminates. An FE buckling analyses of laminated rhombic plates having material nonlinearity, including the Tsai–Wu failure criterion and a nonlinear in-plane shear formulation, was carried out by Hu et al. [48]. Srinivasa et al. [49] examined the buckling loads of skew plates using FE analysis. By considering geometry non-linearity due to excess mechanical deformation in the structure, buckling analysis of composite laminates was studied by Fazzolari et al. [50] using the HSDT. Grover et al. [51] used a shear deformation theory having an inverse hyperbolic function to present a buckling analysis of laminates and sandwich laminates. An exact refined solution procedure for the buckling analysis of thick and thin composite laminates under biaxial loading was presented by Vescovini and Dozion [30]. Fan et al. [52] studied an analytical approach to find buckling load of cylindrical shells under axial compression. Srinivasa et al. [53] presented numerical and experimental studies on buckling of skew laminates with circular cutouts under uniaxial compressive force.

The literature review indicates that few studies on biaxial buckling analysis of laminated composite panel with excision and concentrated mass were carried out and that significant part of the reported studies was based on the FSDT. Due to the complex mechanical behavior of such as structures, which, for example, exhibit complex modes of deformation, their theoretical analysis requires a slightly deeper approach.

In the presented work, an attempt was made to analyze the bi-axial buckling characteristics of laminated composite plates with cutouts and additional mass using a mathematical model (ISDT) which employs a cubic variation of thickness co-ordinate in displacement field. In the present study, an attempt was also made to incorporate shear along with bi-axial force for analyzing the buckling behavior of laminated plates.

## 2. Theory and Formulation

### 2.1. Improved Shear Deformation Theory (ISDT)

In this deformation theory, transverse shear stress at the top and bottom of the laminate are taken as zero. It is assumed that the variation of transverse shear strains is realistic parabolic in shape and use of shear correction factor is hence avoided. The presented theory consists of a realistic cubic variation of in-plane displacement fields. The following equation for displacement fields is being adopted for the presented analysis:

(1) $$\left\{\begin{array}{l} u\\ v\\ w \end{array}\right\}=\left\{\begin{array}{c} u_{0}\\ v_{0}\\ w_{0} \end{array}\right\}+z\left\{\begin{array}{c} \theta_{x}\\ \theta_{y}\\ 0 \end{array}\right\}+z^{2}\left\{\begin{array}{c} \xi_{x}\\ \xi_{y}\\ 0 \end{array}\right\}+z^{3}\left\{\begin{array}{l} \zeta_{x}\\ \zeta_{y}\\ 0 \end{array}\right\}$$

In the equation presented above, *u*, *v* and *w* represent the displacements of a point along the three directions (*x*, *y*, and *z*) respectively, whereas the associated midplane displacements are given by *u*_0_, *v*_0_, and *w*_0_ respectively. *θ_x_* and *θ_y_* signifies the rotations of transverse normal in the *x*-*z* and *y*-*z* planes respectively.

The $\xi_{x},\zeta_{x},\xi_{y}\;\mathrm{and}\;\zeta_{y}$ functions in the above-mentioned equations are determined using an assumption of zero transverse shear strains at the top and bottom surfaces of the plate, i.e.,

(2) $$\gamma_{xz}(x,\;y,\pm h/2)\;and\;\gamma_{yz}\;(x,\;y,\pm h/2)\;=0$$

Since, $\gamma_{xz}=\frac{\delta u}{\delta z}+\frac{\delta w}{\delta x}\;\mathrm{and}\;\gamma_{yz}=\frac{\delta v}{\delta z}+\frac{\delta w}{\delta y}$, So,

(3) $$\gamma_{xz}=\theta_{x}+2z\xi_{x}+3z^{2}\zeta_{x}+\frac{\delta w}{\delta x}.\;\mathrm{and}\;\gamma_{yz}=\theta_{y}+2z\xi_{y}+3z^{2}\zeta_{y}+\frac{\delta w}{\delta y}$$

Using Equations (2) and (3), we obtain

(4) $$\xi_{x}=0\;\mathrm{and}\;\xi_{y}=0,\;\zeta_{x}=-\frac{4}{3h^{2}}(\theta_{x}+\frac{\delta w}{\delta x})\;and\;\zeta_{y}=-\frac{4}{3h^{2}}(\theta_{y}+\frac{\delta w}{\delta y})$$

When replaced, the values obtained in Equation (4) to Equation (1), we get:

(5) $$\left\{\begin{array}{l} u\\ v\\ w \end{array}\right\}=\left\{\begin{array}{l} u_{0}\\ v_{0}\\ w_{0} \end{array}\right\}+z(1-4z^{2}/3h^{2})\left\{\begin{array}{l} \theta_{x}\\ \theta_{y}\\ 0 \end{array}\right\}-4z^{3}/3h^{2}\left\{ \begin{array}{c} \partial w_{0}/\partial x\\ \partial w_{0}/\partial y\\ 0 \end{array}\}\;\mathrm{or},\right.\;\;\left\{\begin{array}{l} u\\ v\\ w \end{array}\right\}=\left\{\begin{array}{l} u_{0}\\ v_{0}\\ w_{0} \end{array}\right\}+z(1-4z^{2}/3h^{2})\left\{\begin{array}{l} \theta_{x}\\ \theta_{y}\\ 0 \end{array}\right\}-4z^{3}/3h^{2}\left\{\begin{array}{c} \psi_{x}{}^{*}\\ \psi_{y}{}^{*}\\ 0 \end{array}\right\}$$

The linear strains may be represented in the form of linear displacement, as follows:

(6) $$\left\{\begin{array}{l} \varepsilon_{x}\\ \varepsilon_{y}\\ \gamma_{xy}\\ \gamma_{xz}\\ \gamma_{yz} \end{array}\right\}=\left\{\begin{array}{c} \partial u/\partial x\\ \partial v/\partial y\\ \partial u/\partial y+\partial v/\partial x\\ \partial u/\partial z+\partial w/\partial x\\ \partial v/\partial z+\partial w/\partial y \end{array}\right\}$$

Using the values of displacement from Equation (5) to Equation (6), the following equation is obtained,

(7) $$\left\{\begin{array}{c} \varepsilon_{x}\\ \varepsilon_{y}\\ \gamma_{xy}\\ \gamma_{xz}\\ \gamma_{yz} \end{array}\right\}=\left\{\begin{array}{c} \partial u_{0}/\partial x\\ \partial v_{0}/\partial y\\ \partial u_{0}/\partial y+\partial v_{0}/\partial x\\ \partial w_{0}/\partial x+\theta_{x}\\ \partial w_{0}/\partial y+\theta_{y} \end{array}\right\}+z(1-4z^{2}/3h^{2})\left\{\begin{array}{c} \partial \theta_{x}/\partial x\\ \partial \theta_{y}/\partial y\\ \partial \theta_{x}/\partial y+\partial \theta_{y}/\partial x\\ 0\\ 0 \end{array}\right\}-\frac{4z^{3}}{3h^{2}}\left\{\begin{array}{c} \partial \psi_{x}{}^{*}/\partial x\\ \partial \psi_{y}{}^{*}/\partial y\\ \partial \psi_{x}{}^{*}/\partial y+\partial \psi_{y}{}^{*}/\partial x\\ 0\\ 0 \end{array}\right\}-\frac{12z^{2}}{3h^{2}}\left\{\begin{array}{c} 0\\ 0\\ 0\\ \theta_{x}+\psi_{x}{}^{*}\\ \theta_{y}+\psi_{y}{}^{*} \end{array}\right\}$$

or

(8) $$\left\{\begin{array}{c} \varepsilon_{x}\\ \varepsilon_{y}\\ \gamma_{xy}\\ \gamma_{xz}\\ \gamma_{yz} \end{array}\right\}=\left\{\begin{array}{c} \varepsilon_{x0}\\ \varepsilon_{y0}\\ \gamma_{xy0}\\ \phi_{x}\\ \phi_{y} \end{array}\right\}+z(1-4z^{2}/3h^{2})\left\{\begin{array}{c} K_{x}\\ K_{y}\\ K_{xy}\\ K_{xz}\\ K_{yz} \end{array}\right\}-\frac{4z^{3}}{3h^{2}}\left\{\begin{array}{c} K^{*}{}_{x}\\ K^{*}{}_{y}\\ K^{*}{}_{xy}\\ K^{*}{}_{xz}\\ K^{*}{}_{yz} \end{array}\right\}-\frac{12z^{2}}{3h^{2}}\left\{\begin{array}{c} K^{**}{}_{x}\\ K^{**}{}_{y}\\ K^{**}{}_{xy}\\ K^{**}{}_{xz}\\ K^{**}{}_{yz} \end{array}\right\}$$

The strains associated with Equation (8) are related to the generalized strains by means of the following expression:

(9) $$\left\{\bar{\varepsilon }\right\}=\left[H\right]\left\{\varepsilon \right\}$$

where $\left\{\bar{\varepsilon }\right\}={\left[\varepsilon_{x}\varepsilon_{y}\gamma_{xy}\gamma_{xz}\gamma_{yz}\right]}^{T}$ and $\left\{\varepsilon \right\}={\left\{\begin{array}{l} \varepsilon_{x0},\kappa_{x},\kappa_{x}^{*},\gamma_{x0},\varepsilon_{y0},\kappa_{y},\kappa_{y}^{*},\gamma_{y0},\\ \kappa_{xy},\kappa_{xy}^{*},w_{0},\theta_{x},\psi_{x},v_{0},\theta_{y},\psi_{y},u_{0} \end{array}\right\}}^{T}$, {ε} is the function of *x* and *y* and [H] is the function of thickness coordinate z.

Further, the strain vector {ε} can be interrelated with displacement vector {*X*} by means of the following relationship.

(10) {ε}=[B]{X}

where $\left\{X\right\}=\left\{u_{0},v_{0},w_{0},\theta_{x},\theta_{y},\psi_{x},\psi_{y}\right\}$.

For the typical lamina (*k*th), the constitutive relations with respect to the material axis can be expressed as: ${\left\{\sigma \right\}}_{k}={\left[Q\right]}_{k}{\left\{\varepsilon \right\}}_{k}$, i.e.,

(11) $${\left\{\begin{array}{c} \sigma_{1}\\ \sigma_{2}\\ \tau_{12}\\ \tau_{13}\\ \tau_{23} \end{array}\right\}}_{k}={\left[\begin{array}{ccccc} Q_{11} & Q_{12} & 0 & 0 & 0\\ Q_{12} & Q_{22} & 0 & 0 & 0\\ 0 & 0 & Q_{66} & 0 & 0\\ 0 & 0 & 0 & Q_{44} & 0\\ 0 & 0 & 0 & 0 & Q_{55} \end{array}\right]}_{k}{\left\{\begin{array}{c} \varepsilon_{1}\\ \varepsilon_{2}\\ \gamma_{12}\\ \gamma_{13}\\ \gamma_{23} \end{array}\right\}}_{k}$$

where, $\frac{\upsilon_{12}}{E_{1}}=\frac{\upsilon_{21}}{E_{2}}$, and

$${\left[\begin{array}{ccccc} Q_{11} & Q_{12} & 0 & 0 & 0\\ Q_{12} & Q_{22} & 0 & 0 & 0\\ 0 & 0 & Q_{66} & 0 & 0\\ 0 & 0 & 0 & Q_{44} & 0\\ 0 & 0 & 0 & 0 & Q_{55} \end{array}\right]}_{k}={\left[\begin{array}{ccccc} E_{1}/1-\nu_{12}\nu_{21} & \nu_{12}E_{2}/1-\nu_{12}\nu_{21} & 0 & 0 & 0\\ \nu_{12}E_{2}/1-\nu_{12}\nu_{21} & E_{2}/1-\nu_{12}\nu_{21} & 0 & 0 & 0\\ 0 & 0 & G_{12} & 0 & 0\\ 0 & 0 & 0 & G_{13} & 0\\ 0 & 0 & 0 & 0 & G_{23} \end{array}\right]}_{k}$$

The stress-strain relationship with respect to global coordinate axis system (*x*, *y*, and *z*) for *k*th lamina can be expressed using the applied transformation coefficients as shown below:

(12) $${\left\{\begin{array}{c} \sigma_{x}\\ \sigma_{y}\\ \tau_{xy}\\ \tau_{xz}\\ \tau_{yz} \end{array}\right\}}_{k}={\left[\begin{array}{ccccc} {\bar{Q}}_{11} & {\bar{Q}}_{12} & 0 & 0 & 0\\ {\bar{Q}}_{12} & {\bar{Q}}_{22} & 0 & 0 & 0\\ 0 & 0 & {\bar{Q}}_{66} & 0 & 0\\ 0 & 0 & 0 & {\bar{Q}}_{44} & 0\\ 0 & 0 & 0 & 0 & {\bar{Q}}_{55} \end{array}\right]}_{k}{\left\{\begin{array}{c} \varepsilon_{x}\\ \varepsilon_{y}\\ \varepsilon_{xy}\\ \gamma_{xz}\\ \gamma_{yz} \end{array}\right\}}_{k}$$

Integration of the stresses through the laminate thickness will help in obtaining the resultant forces and moments acting on the laminate, which is as follows:

$$\left[M\right]=\left[\begin{array}{c} M_{x}\\ M_{y}\\ M_{xy} \end{array}\begin{array}{c} M^{*}{}_{x}\\ M^{*}{}_{y}\\ M^{*}{}_{xy} \end{array}\right]=\sum_{K=1}^{N_{L}}\int_{Z_{K}}^{Z_{K+1}}\left[\begin{array}{c} \sigma_{x}\\ \sigma_{y}\\ \tau_{xy} \end{array}\right]\left[z,z^{3}\right]dz,\;\left[N\right]=\left[\begin{array}{c} N_{x}\\ N_{y}\\ N_{xy} \end{array}\right]=\sum_{K=1}^{N_{L}}\int_{Z_{K}}^{Z_{K+1}}\left[\begin{array}{c} \sigma_{x}\\ \sigma_{y}\\ \tau_{xy} \end{array}\right]dz$$

$$\left[Q,S,S^{*},S^{**}\right]=\left[\begin{array}{cccc} Q_{x} & S_{x} & S_{x}{}^{*} & S_{x}{}^{**}\\ Q_{y} & S_{y} & S_{y}{}^{*} & S_{y}{}^{**} \end{array}\right]=\sum_{K=1}^{N_{L}}\int_{Z_{K}}^{Z_{K+1}}\left[\begin{array}{c} \tau_{xz}\\ \tau_{yz} \end{array}\right]\left[1,z,z^{2},z^{3}\right]dz,$$

or

$$\left\{\bar{\sigma }\right\}=\left[\bar{D}\right]\left\{\bar{\varepsilon }\right\},$$

where

$$\left\{\bar{\sigma }\right\}={\left[N_{x},N_{y},N_{xy},M_{x},M_{y,}M_{xy},M_{x}^{*},M_{y}^{*},M_{xy}^{*},\theta_{x},\theta_{y},S_{x},S_{y},S_{x}^{*},S_{y}^{*},S_{x}^{**},S_{y}^{**}\right]}^{T}$$

$$\left\{\bar{\varepsilon }\right\}={\left[\varepsilon_{x0},\varepsilon_{y0},\gamma_{xy0},K_{x},K_{y,}K_{xy},K_{x}^{*},K_{y}^{*},K_{xy}^{*},\phi_{x},\phi_{y},K_{xz},K_{yz},K_{xz}^{*},K_{yz}^{*}K_{xz}^{**},K_{yz}^{**}\right]}^{T}$$

and the size of the rigidity matrix $\left[\bar{D}\right]$ is 17 × 17.

Thus, by following the standard procedure of FEM the element matrices are assembled which results in global stiffness matrices i.e., [*K*] and [*M*].

### 2.2. Finite Element Formulations

**Figure 1:**
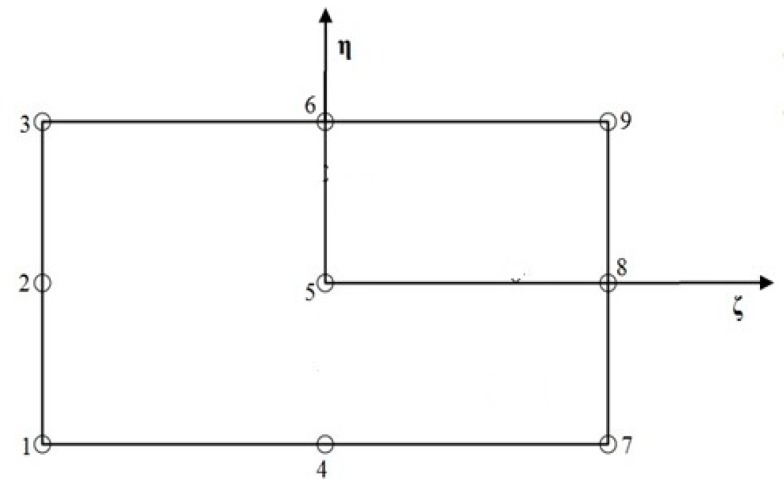
Nine noded curved isoparametric element with typical node numbering.

In this paper, C° isoparametric elements having nine nodes, with seven unknowns per node (Figure 1) i.e., $u_{1}u_{2},u_{3},\psi_{1,}\psi_{2},w_{1}\;\mathrm{and}\;w_{2}$ were used for the proposed finite element model.

The generalized displacements included in the presented theory can be expressed as follows.

(13) $$u_{1}=\sum_{i=1}^{9}N_{i}u_{i};\;u_{2}=\sum_{i=1}^{9}N_{i}v_{i};\;u_{3}=\sum_{i=1}^{9}N_{i}w_{i};\;\psi_{1}=\sum_{i=1}^{9}N_{i}\psi_{1}{}_{i};\;\psi_{2}=\sum_{i=1}^{9}N_{i}\psi_{2}{}_{i};\;w_{1}=\sum_{i=1}^{9}N_{i}w_{1}{}_{i};\;w_{2}=\sum_{i=1}^{9}N_{i}w_{2}{}_{i}$$

In the equation above, the shape function of the related node is represented by $N_{i}$.

Knowing the nodal unknown vector within an element, the mid-surface strains at any point in the plate can be expressed in the matrix form in terms of the global displacements as described below:

(14) $$\left\{\bar{\varepsilon }\right\}=\sum_{i=1}^{9}\left[B_{i}\right]\left\{d_{i}\right\}$$

In the equation shown above, $\left[B_{i}\right]$ is used to express the differential operator matrix of the shape function.

For an element, the element stiffness matrix (say, eth), including the transverse shear effects, flexure and membrane can be given as:

(15) $$\left[K_{e}\right]=\int_{-1}^{1}\int_{-1}^{1}{\left[B\right]}^{T}\left[D\right]\left[B\right]|J|drds$$

(16) $$\left[M_{e}\right]=\int_{-1}^{1}\int_{-1}^{1}{\left[N\right]}^{T}[\rho ]\left[N\right]|J|drds$$

In the above equations, [N] represents the matrix of shape function, [ρ] inertia matrix and |J| represents the determinant of a Jacobian matrix.

For all numerical integrations, a 3 × 3 Gaussian quadrature format was used. Then, the element matrices were grouped together to attain the global stiffness matrices [*K*], in accordance with the typical procedure of the FE method as considered by Bathe [54].

The C_0_ FE formulation although being a 2D solutions allowed to obtain the results closer to 3D elastic solutions. This helped to reduce the complex solutions to a simpler form.

### 2.3. Buckling Analysis

The buckling of laminated composite plates was carried out using the above-mentioned theory and FE formulation to study the laminated plates with cutout and concentrated mass. The geometric stiffness matrix required in the buckling analysis can be derived as explained below. For this purpose, the nonlinear strain vector/geometric strain vector may be extracted as:

(17) $$\left\{\varepsilon_{G}\right\}=\left[\begin{array}{l} 1/2(\partial w/\partial x{)}^{2}+1/2(\partial u/\partial x{)}^{2}+1/2(\partial v/\partial x{)}^{2}\\ 1/2(\partial w/\partial y{)}^{2}+1/2(\partial u/\partial y{)}^{2}+1/2(\partial v/\partial y{)}^{2}\\ (\partial w/\partial x)(\partial w/\partial y)+(\partial u/\partial x)(\partial u/\partial y)+(\partial v/\partial x)(\partial v/\partial y) \end{array}\right]\;\mathrm{or}\;\left\{\varepsilon_{G}\right\}=1/2\left[A_{G}\right]\left\{\theta \right\}$$

where, {θ}=[∂w/∂x  ∂w/∂y ∂u/∂x ∂u/∂y ∂v/∂x ∂v/∂y] and

$$\left[A_{G}\right]=\left[\begin{array}{cccccc} \partial w/\partial x & 0 & \partial u/\partial x & 0 & \partial v/\partial x & 0\\ 0 & \partial w/\partial y & 0 & \partial u/\partial y & O & \partial w/\partial y\\ \partial w/\partial y & \partial w/\partial x & \partial u/\partial y & \partial u/\partial x & \partial v/\partial y & \partial v/\partial x \end{array}\right]$$

Further,

(18) $$\left\{\theta \right\}=\left[H_{G}\right]\left\{\varepsilon \right\}=\left[H_{G}\right][B]\{\delta \}$$

where the matrix [*H_G_*] is the function of thickness coordinate z.

The geometric stiffness matrix [*K_Ge_*] of an element can be developed using Equations (15) and (18) and it may be stated as:

(19) $$\left[K_{Ge}\right]=\sum_{k=1}^{n_{u}+n_{l}}∭{\left[\varepsilon \right]}^{T}[S^{i}]\;[\varepsilon ]\;dx\;dy\;dz\;=\sum_{k=1}^{n_{u}+n_{l}}∭{\left[B\right]}^{T}{\left[H_{G}\right]}^{T}[S^{i}][H_{G}][B]\;dx\;dy\;dz\;=\;\frac{1}{2}\;\iint {\left\{\varepsilon \right\}}^{T}\left[G\;\right]\left\{\varepsilon \right\}dx\;dy$$

where,

(20) $$\left[G\right]=\overset{n_{u}+n_{l}}{\underset{k=1}{\sum }}\int^{\text{​}}{\left[H_{G}\right]}^{T}\left[S^{i}\right]\left[H_{G}\right]dz$$

and $\left[S^{i}\right]$ is in-plane stress components of the *i*-th layer.

$$\left[S^{i}\right]=\left[\begin{array}{cccccc} \sigma_{xx} & \tau_{xy} & 0 & 0 & 0 & 0\\ \tau_{xy} & \sigma_{yy} & 0 & 0 & 0 & 0\\ 0 & 0 & \sigma_{xx} & \tau_{xy} & 0 & 0\\ 0 & 0 & \tau_{xy} & \sigma_{yy} & 0 & 0\\ 0 & 0 & 0 & 0 & \sigma_{xx} & \tau_{xy}\\ 0 & 0 & 0 & 0 & \tau_{xy} & \sigma_{yy} \end{array}\right]$$

Hence the final governing equation for buckling analysis may be written as:

(21) $$\left\{\left[K\right]-\lambda {\left[K\right]}_{G}\right\}\left\{\delta \right\}=\left\{0\right\}$$

where {*δ*} is the nodal displacement vector, *λ* is the critical buckling load and [*K*], [*K*]*_G_* are the linear and geometric stiffness matrices, respectively.

## 3. Numerical Application and Results

### 3.1. Boundary Conditions

The boundary conditions mainly applied to the subsequent examples are, simply supported (SSSS) and clamped boundary conditions (CCCC) which include:

SSSS:

$u_{2}=u_{3}=\psi_{2}=w_{2}=0$ at *x* = 0, a
$u_{1}=u_{3}=\psi_{1}=w_{1}=0$ at *y* = 0, b

CCCC:

$u_{1}=u_{2}=u_{3}=\psi_{1}=\psi_{2}=w_{1}=w_{2}=0$ at *x* = 0, a and at *y* = 0, b
Other than these, four more boundary conditions are used in different examples:
CCFF = Clamped, Clamped, Fixed, Fixed
CFCF = Clamped, Fixed, Clamped, Fixed
SSCC = Simply supported, Simply supported, Clamped, Clamped
CCSS = Clamped, Clamped, Simply supported, Simply supported.

### 3.2. Engineering Properties

For all further investigations, unless mentioned otherwise, the composites with following properties were taken *E*_1_ = 25*E*_2_, *E*_2_ =1, *G*_12_ = *G*_13_ = 0.5*E*_2_, *G*_23_ = 0.2*E*_2_, *ρ* = 1 and *ν*_12_ = 0.25, after Chakrabarti and Sheikh [46] as the common standard values for these materials. The non-dimensional buckling load for composites were taken as $\bar{\lambda }=(\lambda b^{2}/E_{2}h^{3})$. The value of additional mass is given by the following equation: $\bar{\underset{˙}{M}}=\frac{M}{\rho ha^{2}}$.

### 3.3. Convergence and Comparison Studies

**Figure 2:**
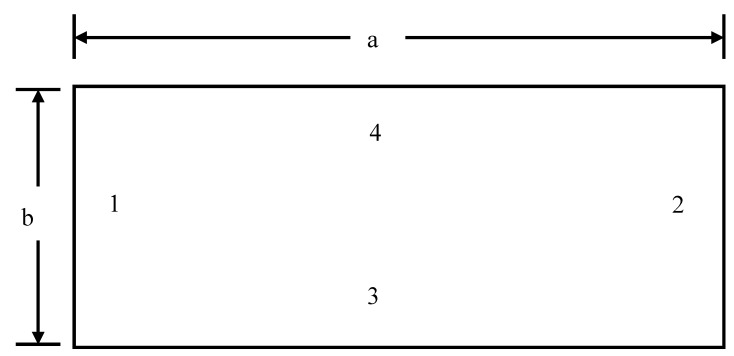
Numbering of edges of the plate.

Convergence study was done to determine the required mesh size N × N at which the dimensionless buckling load values converged. It may be concluded from Table 1, that the values of non-dimensional buckling load converged at N = 20. Therefore, for all subsequent analyses mesh size of 20 × 20 (full) was taken into consideration. The numbering of edges in the plate is shown in Figure 2.

It is clear from the literature review that based upon present theory, for the buckling analysis of the laminated composite plates having mass variation in the form of cutout and concentrated mass no results are available. Therefore, in order to show the efficiency of the present FE model the obtained results were evaluated with the results published by Reddy and Phan [41], Pandit et al. [55], Liu et al. [56], and Singh et al. [57] based upon FSDT, HSDT, GRBF (Gaussian radial basic function) and MQRBF (Multiquadric radial basic function).

Analysis of cross-ply (0°/90°/90°/0°) and (0°/90°/0°) square laminates under the effect of uni-axial compression was carried in this example and shown in Table 2. In this example, the analysis of a full plate was done with *a*/*h* ratio equal to 10. Table 2 shows, the utility of the present FE model in predicting the non-dimensional buckling load close to the analytical results from the previously quoted literature.

In another problem, a square laminated composite plate having lamination scheme as (0°/90°/0°) was analyzed for different values of elastic modulus ratio i.e., *E*_1_/*E*_2_. The results were compared with the results of Vesconi and Dozio [30] as shown in Table 3.

The aspect ratios (*a*/*h*) taken into consideration for the study were 10, 20 and 50. Table 3 shows that for both ratios of N_x_/N_y_ the results are in good accordance with those reported by Vescovini and Dozio [30]. Buckling load for two sides clamped laminates compared to experimental and FEM (ANSYS) results of Baba [58] are shown in Table 4.

### 3.4. Novel Results

**Figure 3:**
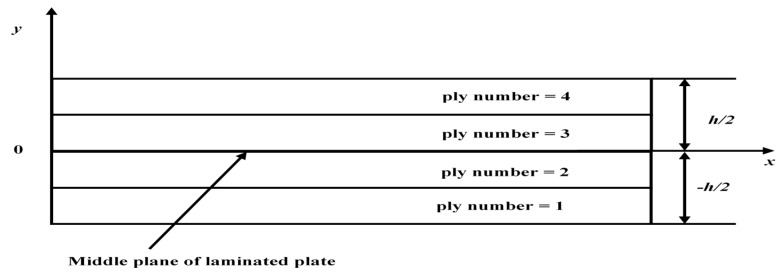
Typical front view of a four-layer laminated plate with ply numbering.

After validating the presented FE model based upon the above-mentioned theory through comparison studies, new problems were worked out to analyze the effect of openings and additional mass on buckling of laminated composite plates. For the following examples of buckling analyses, various laminated composite plates with different lamination schemes and boundary conditions were considered. The lamination schemes adopted for the various problems are given in Table 5. The geometrical and material parameters adopted for the present analyses were assumed as defined in the previous section. The ply numbering scheme shown in Figure 3, is in such a way that the counting of the lamina was done from bottom to top. In the present examples, the laminated composite plates are considered having the additional mass ($\bar{M}=\frac{M}{\rho ha^{2}}$ = 0.5, 1 and 2) and square cutout (0.2a, 0.4a and 0.6a), concentrated at the center.

#### 3.4.1. Laminated Composite Plates with Additional Mass

**Figure 4:**
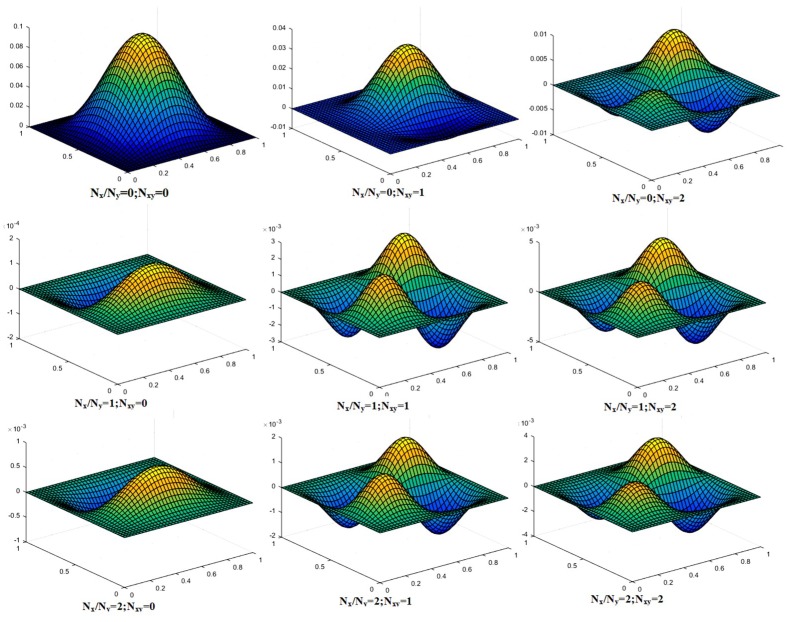
Mode shape for a laminated composite plate with lamination scheme A, *a*/*h* = 100, $\bar{M}=0.05$ and CCCC boundary condition.

**Figure 5:**
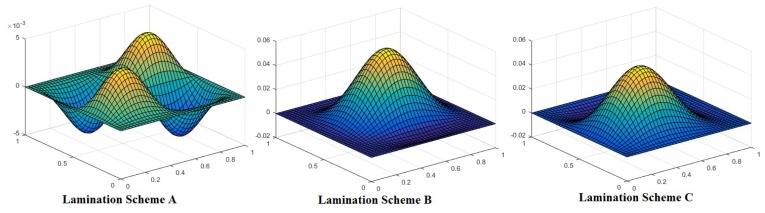
Mode shape for laminated composite plates with *a*/*h* = 20, $\bar{M}=1$, N_x_/N_y_ = 2, N_xy_ = 2 and CCCC boundary condition.

**Figure 6:**
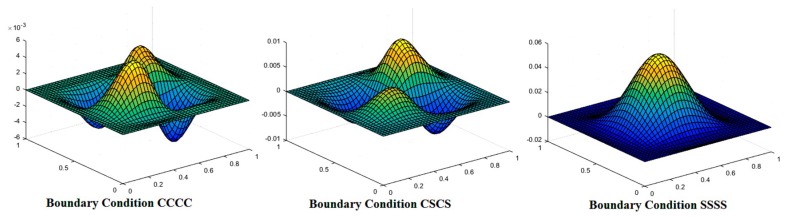
Mode shape for laminated composite plate with lamination scheme C, *a*/*h* = 5, N_x_/N_y_ = 1, N_xy_ = 1 and $\bar{M}=2$.

Many novel problems were solved and shown in Table 6, Table 7 and Table 8 with variation in values of aspect ratio (*a*/*h*), nature of loading (N_x_/N_y_ & N_xy_), additional mass $\bar{M}$ (0.05, 1 and 2), lamination schemes and boundary conditions. Further, the mode shapes of plates were also shown in Figure 4, Figure 5 and Figure 6. In all the preceding problems the material properties were taken as defined in the previous section and the additional mass was concentrated at the central node.

In Table 6a,b, the non-dimensional buckling loads for composite plates having lamination scheme A and different boundary conditions are shown. In the present problem, different values of aspect ratio (*a*/*h* = 100, 20 and 5) and N_x_/N_y_ (N_x_/N_y_ = 0, 1 and 2) are taken in consideration. The values of shear forces (N_xy_) are taken as 0, 1 and 2. It is observed from the table that the non-dimensional buckling loads are minimum for lower values of *a*/*h* and higher values of biaxial (N_x_/N_y_) and shear (N_xy_) forces. The buckling load on plates is proportional to h^2^ and buckling load should decrease with a decrease in thickness. But the presented results are in terms of the non-dimensional buckling load parameter (*λ* = N_x_ *a*^2^/(E^2^*h*^3^)) which is inversely proportional to cubed thickness. Hence, the non-dimensional buckling load parameter increases with a decrease in thickness. It may be also observed that for laminated plates having *a*/*h* ratio greater than 5, the CCCC boundary condition has the greater values of non-dimensional buckling loads (this is due to the highest stiffness of CCCC boundary conditions) whereas the lowest for CFCF boundary condition. Whereas for an *a*/*h* ratio equal to 5, any loading condition has almost the same nondimensional buckling load for all boundary conditions except for CFCF boundary condition. The nondimensional buckling load was also found to have the least variation for increasing values of additional mass for a particular aspect ratio and applied loading conditions. In Table 7a,b, and Table 9a,b, the other problems were solved for the composite plates having lamination scheme B and C with different boundary conditions. In the present problem, different values of aspect ratio (*a*/*h* = 100, 20 and 5) and N_x_/N_y_ (N_x_/N_y_ = 0, 1 and 2) were taken in consideration. The values of shear forces (N_xy_) were taken as 0, 1 and 2. In the tables, all trends of variations for non-dimensional buckling load with respect to different loading and boundary conditions are similar to the ones presented in Table 6a,b. It may be concluded from the tables that for any boundary condition different than CFCF and CCFF as the number of plies are increased the values of non-dimensional buckling load increases along with any particular value of aspect ratio, loading condition and additional mass.

The mode shapes for the buckled laminated composite plates are shown in Figure 4, Figure 5 and Figure 6. Figure 4 shows mode shapes for composite plates having lamination scheme A and clamped at all edges. The value of additional mass and *a*/*h* ratio was taken as 0.05 and 100, respectively. The different conditions of uni-axial and bi-axial loading with or without shear were taken into consideration. Figure 4 shows that mode shapes for bi-axial loading are different from uni-axial buckling whereas mode shapes for different values of bi-axial loading remain the same, only the values are changed. Figure 5 shows mode shapes for different lamination schemes keeping other conditions like *a*/*h* ratio, nature of the load, the values of additional mass and boundary conditions are constant. It can be seen that on changing the lamination scheme, the mode shape changes; this may be due to the fact that a different orientation of fiber in plies affects the strength of laminate and thereby changing the mode of deformation.

Figure 6 presents the variation of mode shape for different boundary conditions. The figure indicates that mode shapes are affected by variation in the boundary condition. The least distorted mode shape is observed for SSSS boundary condition.

#### 3.4.2. Laminated Composite Plates with Central Cutout

**Figure 7:**
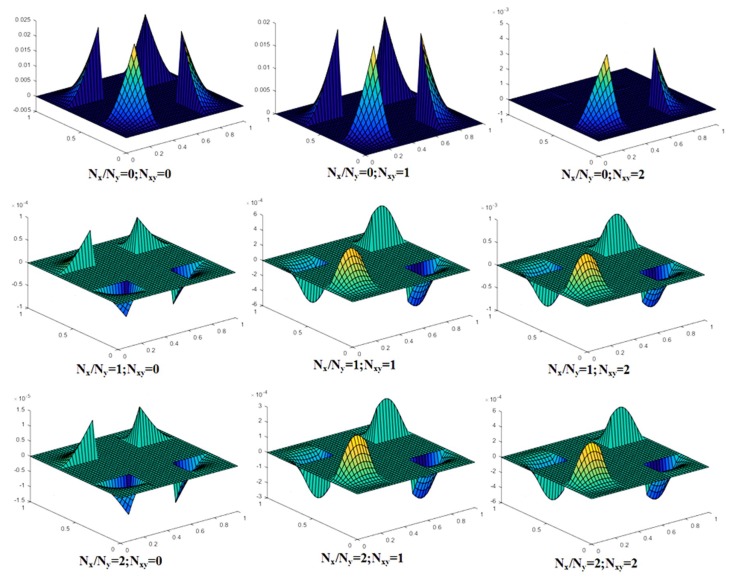
Mode shape for a laminated composite plate with lamination scheme A, *a*/*h* = 100, cutout size 0.4a and CCCC boundary condition.

**Figure 8:**
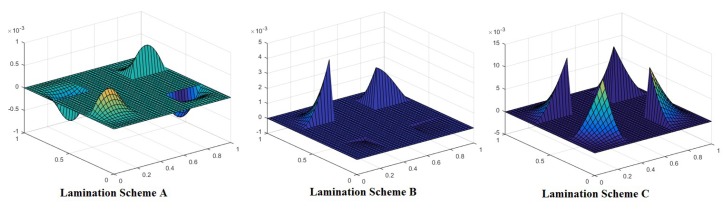
Mode shape for a laminated composite plate with lamination scheme B, *a*/*h* = 20, cutout size 0.4a, N_x_/N_y_ = 2, N_xy_ = 2 and CCCC boundary condition.

**Figure 9:**
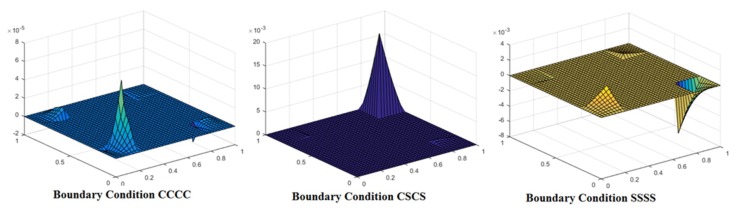
Mode shape for laminated composite plate with lamination scheme C, a/h = 5, cutout size 0.6a and N_x_/N_y_ = 1, N_xy_ = 1.

Many novel problems were solved and shown in Table 9, Table 10 and Table 11 taking different values of aspect ratio (*a*/*h*), nature of loading (N_x_/N_y_ & N_xy_), cutout sizes (0.2a × 0.2a, 0.4a × 0.4a and 0.6a × 0.6a), lamination schemes and boundary conditions. Further, the mode shapes were also drawn and shown in Figure 7, Figure 8 and Figure 9. In all the preceding problems the material properties were assumed as defined in the previous section and a square cutout is taken at the center of the plate

In Table 9a,b, the non-dimensional buckling loads for the composite plates having lamination scheme A and different boundary conditions are shown. In the present problem, the different values of aspect ratio (*a*/*h* = 100, 20 and 5) and N_x_/N_y_ (N_x_/N_y_ = 0, 1 and 2) were taken in consideration. The values of shear forces (N_xy_) were taken as 0, 1 and 2. The table shows that for lower values of aspect ratio the non-dimensional buckling load is minimum. The values of loads are also found to be lower for higher values of biaxial (N_x_/N_y_) and shear (N_xy_) forces. It may be also observed, that for laminated plates having the *a*/*h* ratio greater than 5, CCCC boundary conditions have the greater values of the non-dimensional buckling loads whereas the lowest is for CFCF boundary conditions. Whereas for *a*/*h* ratio equal to 5, any loading condition has almost the same nondimensional buckling load for all the boundary conditions except for CFCF boundary condition. The non-dimensional buckling load is found to increase along with the size of the central cutout area.

In Table 10 and Table 11, other solved problems are presented for composite plates having lamination scheme B and C with different boundary conditions. In the present problem, different values of aspect ratio (*a*/*h* = 100, 20 and 5) and N_x_/N_y_ (N_x_/N_y_ = 0, 1 and 2) were taken in consideration. The values of shear forces (N_xy_) were taken as 0, 1 and 2. In the tables, all trends of variations for non-dimensional buckling load with respect to different loading and boundary conditions were similar to the ones presented in Table 9. The values presented in the discussed tables show that for any boundary condition the values of non-dimensional buckling load increase along with the number of plies for any particular value of aspect ratio, loading condition and cutout size.

The mode shapes for the central area of buckled laminated composite plates are shown in Figure 7, Figure 8 and Figure 9. The plane section area with no deformations represents the cutout in the plate. As there is no material at the cutout in the plate, it does not move in the mode shape. Figure 8 shows mode shapes for central area of composite plates clamped at all edges and having lamination scheme A. The value of a central square cutout and *a*/*h* ratio were taken as 0.4a and 100, respectively. The different conditions of uni-axial and bi-axial loading with or without shear were taken into consideration. From Figure 7, it can be seen that mode shapes for bi-axial loading are different from uni-axial buckling, whereas the mode shapes for different values of bi-axial loading remain same, only the values are different.

Figure 8 shows the mode shapes for different lamination schemes obtained preserving other conditions, like *a*/*h* ratio, loading condition, cutout size and boundary conditions constant. It is visible that changes in lamination scheme trigger changes in the mode shapes, this may be due to the fact that the different orientation of fiber in plies affects the strength of laminate and thereby changing the mode of deformation.

Figure 9 shows the variation of mode shape for different boundary conditions keeping other parameters, like cutout size, aspect ratio and nature of loading, constant. The figure indicates that mode shapes are affected by variation in the boundary conditions.

## 4. Conclusions

In this paper, using the presented ISDT formulation and C° finite element model a computer code for Uni-axial and Bi-axial buckling analysis of laminated composite plates were developed. The proposed FE model based on the presented theory was analyzed for the first time. Along with this theory, the transverse shear stress continuity was also incorporated at the interface of each layer in addition to zero transverse shear stress at the top and bottom of the plate. The performed analyses showed that the obtained results are much improved over the other existing models (FSDT and HSDT). Various novel problems with different geometrical properties, loading, and boundary conditions and ply orientation were analyzed for the laminated composite plates. The following conclusions were drawn from the presented study:

1. A relative study of the results from literature and present mathematical formulation shows that the obtained novel formulation gives good results.
2. The dimensionless value of buckling load was found to be decreasing along with the increase in the values of N_x_/N_y_ and N_xy_.
3. It was observed that for any value of the applied additional mass and cutout, the non-dimensional buckling load was minimum for the minimum aspect ratio (*a*/*h*), and as the aspect ratio decreases the variation of non-dimensional buckling load with respect to the applied boundary conditions becomes insignificant for the laminated composite plates.
4. It can be seen that for any applied aspect ratio, the CCCC boundary condition had the highest values of non-dimensional buckling load whereas CCFF had the lowest for laminated composite plates having additional mass at the central node. It was also concluded that for higher lamination plies the value for buckling loads was higher, irrespective of any boundary and loading conditions.
5. It was concluded from the presented study that mode shapes for bi-axial loading were different from the uni-axial buckling whereas for different values of bi-axial loading the mode shape remains the same despite the different observed values. The mode shape was also found to vary for change in the applied boundary conditions. These conditions were the same for both the cases i.e., for additional mass and cutout.
6. It can be seen that on changing lamination scheme, with the remaining parameters assumed as constant, the mode shape changed, and this may be due to the fact that the different orientation of fiber in plies affects the strength of laminate, thereby changing the mode of deformation. The condition was the same for additional mass and cutouts.

## Figures and Tables

**Table 1:** Convergence study of nondimensional buckling load $\bar{\lambda }=(\lambda b^{2}/E_{2}h^{3})$ with *a*/*h* for a simply supported cross-ply square laminated plate (0°/90°/90°/0°)_t_ with material properties: *E*_1_/*E*_2_ = 40, *G*_12_ = *G*_23_ = 0.6*E*_2_, *G*_23_ = 0.5*E*_2_, *ν*_12_ = 0.25.

References	Theory	Thickness Ratio (*a*/*h*)			
		100	50	20	10
Present (8 × 8)	ISDT	36.5865	35.8502	31.9649	23.3151
Present (10 × 10)	ISDT	36.3277	35.6464	31.8054	23.2024
Present (16 × 16)	ISDT	36.1909	35.4535	31.6393	23.0835
Present (18 × 18)	ISDT	36.1925	35.4327	31.6181	23.0678
Present (20 × 20)	ISDT	36.1918	35.4293	31.6024	23.0565
Present (22 × 22)	ISDT	36.1919	35.4294	31.6024	23.0566

**Table 2:** Validation of nondimensional buckling load $\bar{\lambda }=(\lambda b^{2}/E_{2}h^{3})$ for a uni-axial buckling of simply supported cross-ply square laminated plate: *G*_12_ = *G*_23_ = 0.6*E*_2_, *G*_23_ = 0.5*E*_2_, *ν*_12_ = 0.25 and *a*/*h* = 10.

Lamination Scheme	Source	*E*_1_/*E*_2_				
		3	10	20	30	40
(0°/90°/0°)	Present	5.3142	9.6982	14.6927	18.6343	21.8415
	Reddy and Phan (HSDT) [41]	5.3933	9.9406	15.2980	19.6740	23.3400
	Reddy and Phan (FSDT) [41]	5.3931	9.9625	15.3510	19.7560	23.4530
	Singh et al. (GRBF) [57]	5.3958	9.8487	14.9411	18.9861	22.3049
	Singh et al. (MQRBF) [57]	5.4108	9.8956	15.0326	19.1227	22.4881
(0°/90°/90°/0°)	Present	5.3197	9.8087	15.1025	19.4295	23.0565
	Nguyen-Van et al. [23]	5.3210	9.8090	15.0640	19.3390	22.9120
	Liu et al. [56]	5.4120	10.013	15.3090	19.7780	23.4120
	Pandit et al. [55]	5.3290	9.8204	15.1314	19.4774	23.1214
	Singh et al. (GRBF) [57]	5.3991	9.9527	15.3343	19.7490	23.4668
	Singh et al. (MQRBF) [57]	5.4161	10.025	15.5252	20.0520	23.8166

**Table 3:** Validation of nondimensional buckling load $\bar{\lambda }=(\lambda b^{2}/E_{2}h^{3})$ for a bi-axial buckling (N_x_/N_y_) of simply supported (0°/90°/0°) cross-ply square laminated plate: *G*_12_ = *G*_23_ = 0.6*E*_2_, *G*_23_ = 0.5*E*_2_, *ν*_12_ = 0.25.

N_x_/N_y_	E_1_/E_2_	Source	*a*/*h*		
			10	20	50
1	10	Present	4.8441	5.4890	5.7084
		Vescovini and Dozio [30]	4.9095	5.5082	5.7063
	25	Present	7.9066	10.0852	10.7040
		Vescovini and Dozio [30]	8.6820	10.8768	11.7320
2	10	Present	6.4611	7.3211	7.6138
		Vescovini and Dozio [30]	6.5461	7.3442	7.6084
	25	Present	11.1735	14.3514	15.6099
		Vescovini and Dozio [30]	11.5760	14.5025	15.6426

**Table 4:** Buckling load of two side clamped (CC) laminates.

Lamination Angle	*a/h = 100*			*a/h = 50*		
	Baba [58] *	Baba [58] ^#^	Present	Baba [58] *	Baba [58] ^#^	Present
[90]_8_	101.00	106.33	102.72	356.00	425.52	408.75
[(0/90)_2_]_s_	319.00	366.52	368.45	1105.00	1455.50	1455.93
[(0/90)_2_]_as_	260.00	290.22	290.39	844.00	1154.50	1149.00

* Experimental; ^#^ Ansys.

**Table 5:** Plate lamination schemes.

Symbol	Lamination Scheme *
A	(0°/90°/0°)_t_
B	(−45°/45°/−45°/45°)_t_
C	(45°/0°/−45°/0°/−45°/90°/0°/45°/0°/90°/−45°/0°/−45°/0°/45°)_t_

* t = total laminate.

**Table 6:** (**a**) Non-dimensional buckling load $\bar{\lambda }=(\lambda b^{2}/E_{2}h^{3})$ for a bi-axial buckling of a composite plate having lamination scheme A and additional mass; (**b**) Non-dimensional buckling load $\bar{\lambda }=(\lambda b^{2}/E_{2}h^{3})$ for a bi-axial buckling of a composite plate having lamination scheme A and additional mass.

(**a**)										
***a/h***	**Concentrated Mass**	**CCCC**								
		**N_x_/N_y_ = 0**			**N_x_/N_y_ = 1**			**N_x_/N_y_ = 2**		
		**N_xy_ = 0**	**N_xy_ = 1**	**N_xy_ = 2**	**N_xy_ = 0**	**N_xy_ = 1**	**N_xy_ = 2**	**N_xy_ = 0**	**N_xy_ = 1**	**N_xy_ = 2**
100	0.50	86.43	53.85	32.08	27.75	24.34	19.47	15.24	14.58	13.15
	1.00	86.21	53.81	32.09	27.75	24.34	19.47	15.24	14.58	13.15
	2.00	85.77	53.72	32.09	27.75	24.34	19.47	15.24	14.58	13.15
20	0.50	53.07	27.78	16.89	18.76	15.22	11.45	10.25	9.53	8.20
	1.00	52.94	27.76	16.88	18.76	15.22	11.45	10.25	9.53	8.20
	2.00	52.68	27.72	16.87	18.76	15.22	11.45	10.25	9.53	8.20
5	0.50	7.76	2.78	1.59	1.62	1.36	1.04	0.82	0.79	0.71
	1.00	7.76	2.78	1.59	1.62	1.36	1.04	0.82	0.79	0.71
	2.00	7.76	2.78	1.59	1.62	1.36	1.04	0.82	0.79	0.71
		**CCSS**								
100	0.50	81.18	50.07	30.40	24.55	22.07	18.21	12.87	12.51	11.60
	1.00	81.01	50.08	30.40	24.54	22.07	18.21	12.86	12.51	11.60
	2.00	80.68	50.09	30.40	24.53	22.05	18.20	12.86	12.51	11.60
20	0.50	48.64	26.45	15.81	16.24	13.81	10.62	9.12	8.62	7.57
	1.00	48.54	26.45	15.81	16.24	13.81	10.62	9.12	8.62	7.57
	2.00	48.35	26.45	15.81	16.24	13.82	10.62	9.12	8.62	7.57
5	0.50	7.43	2.77	1.59	1.62	1.36	1.04	0.81	0.79	0.71
	1.00	7.42	2.77	1.59	1.62	1.36	1.04	0.81	0.79	0.71
	2.00	7.40	2.77	1.59	1.62	1.36	1.04	0.81	0.79	0.71
		**SSCC**								
100	0.50	30.57	23.89	16.97	13.18	12.30	10.60	8.20	7.95	7.38
	1.00	30.49	23.86	16.96	13.16	12.29	10.60	8.19	7.95	7.38
	2.00	30.34	23.78	16.92	13.13	12.26	10.58	8.18	7.94	7.37
20	0.50	26.17	18.50	12.35	11.29	10.14	8.29	7.02	6.69	6.00
	1.00	26.11	18.47	12.34	11.27	10.13	8.28	7.01	6.68	5.99
	2.00	25.98	18.42	12.33	11.25	10.12	8.27	7.00	6.67	5.99
5	0.50	7.51	2.77	1.59	1.62	1.36	1.04	0.82	0.79	0.70
	1.00	7.51	2.77	1.59	1.62	1.36	1.04	0.82	0.79	0.70
	2.00	7.50	2.77	1.59	1.62	1.36	1.04	0.82	0.79	0.70
(**b**)										
***a/h***	**Concentrated Mass**	**CCFF**								
		**N_x_/N_y_ = 0**			**N_x_/N_y_ = 1**			**N_x_/N_y_ = 2**		
		**N_xy_ = 0**	**N_xy_ = 1**	**N_xy_ = 2**	**N_xy_ = 0**	**N_xy_ = 1**	**N_xy_ = 2**	**N_xy_ = 0**	**N_xy_ = 1**	**N_xy_ = 2**
100	0.50	78.55	40.13	23.86	13.06	12.49	11.24	6.78	6.69	6.47
	1.00	78.47	40.11	23.86	13.06	12.49	11.24	6.78	6.69	6.47
	2.00	78.28	40.09	23.85	13.06	12.49	11.24	6.78	6.69	6.47
20	0.50	46.41	20.91	12.31	8.91	8.37	7.16	4.70	4.61	4.39
	1.00	46.37	20.90	12.31	8.91	8.37	7.16	4.70	4.61	4.39
	2.00	46.27	20.89	12.30	8.91	8.37	7.16	4.70	4.61	4.39
5	0.50	6.59	2.71	1.55	1.60	1.34	1.01	0.81	0.79	0.70
	1.00	6.58	2.71	1.55	1.60	1.34	1.01	0.81	0.79	0.70
	2.00	6.57	2.71	1.55	1.60	1.34	1.01	0.81	0.79	0.70
		**CFCF**								
100	0.50	6.52	2.29	1.34	2.62	1.48	1.02	1.42	1.04	0.80
	1.00	6.52	2.29	1.34	2.62	1.48	1.02	1.42	1.04	0.80
	2.00	6.52	2.29	1.34	2.62	1.48	1.02	1.42	1.04	0.80
20	0.50	5.71	1.99	1.17	2.31	1.30	0.89	1.26	0.91	0.70
	1.00	5.71	1.99	1.17	2.31	1.30	0.89	1.26	0.91	0.70
	2.00	5.71	1.99	1.17	2.31	1.30	0.89	1.26	0.91	0.70
5	0.50	3.42	1.17	0.68	1.28	0.75	0.51	0.69	0.52	0.40
	1.00	3.42	1.17	0.68	1.28	0.75	0.51	0.69	0.52	0.40
	2.00	3.42	1.17	0.68	1.28	0.75	0.51	0.69	0.52	0.40
		**SSSS**								
100	0.50	23.72	20.27	15.44	10.62	10.07	8.91	5.90	5.80	5.54
	1.00	23.67	20.24	15.42	10.62	10.07	8.91	5.90	5.80	5.54
	2.00	23.58	20.18	15.39	10.62	10.07	8.91	5.90	5.80	5.54
20	0.50	20.02	15.92	11.35	9.37	8.60	7.22	5.15	5.02	4.69
	1.00	19.98	15.90	11.34	9.38	8.60	7.22	5.15	5.02	4.69
	2.00	19.90	15.86	11.32	9.39	8.60	7.22	5.15	5.02	4.69
5	0.50	6.69	2.76	1.58	1.62	1.36	1.04	0.82	0.79	0.71
	1.00	6.67	2.76	1.58	1.62	1.36	1.04	0.82	0.79	0.71
	2.00	6.65	2.76	1.58	1.62	1.36	1.04	0.82	0.79	0.71

**Table 7:** (**a**) Non-dimensional buckling load $\bar{\lambda }=(\lambda b^{2}/E_{2}h^{3})$ for a bi-axial buckling of a composite plate having lamination scheme B and additional mass; (**b**) Non-dimensional buckling load $\bar{\lambda }=(\lambda b^{2}/E_{2}h^{3})$ for a bi-axial buckling of a composite plate having lamination scheme B and additional mass.

**(a)**										
***a/h***	**Concentrated Mass**	**CCCC**								
		**N_x_/N_y_ = 0**			**N_x_/N_y_ = 1**			**N_x_/N_y_ = 2**		
		**N_xy_ = 0**	**N_xy_ = 1**	**N_xy_ = 2**	**N_xy_ = 0**	**N_xy_ = 1**	**N_xy_ = 2**	**N_xy_ = 0**	**N_xy_ = 1**	**N_xy_ = 2**
100	0.50	85.59	56.96	37.76	43.51	36.63	28.24	28.43	26.06	22.04
	1.00	85.37	56.89	37.74	43.45	36.59	28.23	28.40	26.04	22.03
	2.00	84.95	56.76	37.69	43.33	36.53	28.20	28.35	26.01	22.00
20	0.50	58.81	34.48	21.93	31.12	23.59	17.12	19.77	17.15	13.74
	1.00	58.68	34.45	21.92	31.08	23.58	17.12	19.75	17.14	13.73
	2.00	58.43	34.40	21.90	30.99	23.54	17.10	19.72	17.12	13.72
5	0.50	8.44	2.97	1.72	2.10	1.64	1.19	1.08	1.01	0.86
	1.00	8.43	2.97	1.72	2.10	1.64	1.19	1.08	1.01	0.86
	2.00	8.42	2.97	1.72	2.10	1.64	1.19	1.08	1.01	0.86
		**CCSS**								
100	0.50	63.09	47.19	32.86	35.76	31.53	25.10	21.48	20.41	18.18
	1.00	62.96	47.14	32.84	35.72	31.50	25.09	21.48	20.41	18.18
	2.00	62.71	47.03	32.79	35.64	31.44	25.06	21.48	20.41	18.18
20	0.50	45.00	30.00	19.82	25.39	20.87	15.64	15.64	14.69	12.45
	1.00	44.91	29.98	19.81	25.36	20.85	15.63	15.64	14.70	12.45
	2.00	44.73	29.92	19.79	25.30	20.82	15.62	15.64	14.72	12.46
5	0.50	7.97	2.96	1.71	2.10	1.62	1.21	1.07	1.01	0.86
	1.00	7.97	2.96	1.71	2.10	1.62	1.21	1.07	1.01	0.86
	2.00	7.97	2.96	1.71	2.10	1.62	1.21	1.07	1.01	0.86
		**SSCC**								
100	0.50	54.41	39.17	26.76	23.98	21.79	18.10	15.29	14.65	13.24
	1.00	54.27	39.12	26.74	23.96	21.77	18.09	15.28	14.64	13.23
	2.00	54.00	39.01	26.70	23.90	21.73	18.06	15.26	14.62	13.22
20	0.50	42.54	27.86	18.21	19.20	16.68	13.14	12.26	11.48	9.96
	1.00	42.43	27.82	18.20	19.18	16.66	13.13	12.25	11.47	9.96
	2.00	42.23	27.76	18.18	19.14	16.64	13.11	12.23	11.46	9.95
5	0.50	8.08	2.96	1.71	2.10	1.64	1.19	1.08	1.01	0.86
	1.00	8.08	2.96	1.71	2.10	1.64	1.19	1.08	1.01	0.86
	2.00	8.08	2.96	1.71	2.10	1.64	1.19	1.08	1.01	0.86
**(b)**										
***a/h***	**Concentrated Mass**	**CCFF**								
		**N_x_/N_y_ = 0**			**N_x_/N_y_ = 1**			**N_x_/N_y_ = 2**		
		**N_xy_ = 0**	**N_xy_ = 1**	**N_xy_ = 2**	**N_xy_ = 0**	**N_xy_ = 1**	**N_xy_ = 2**	**N_xy_ = 0**	**N_xy_ = 1**	**N_xy_ = 2**
100	0.50	55.60	35.20	22.84	18.21	16.78	14.16	9.98	9.73	9.12
	1.00	55.55	35.20	22.84	18.21	16.78	14.16	9.98	9.73	9.11
	2.00	55.43	35.20	22.84	18.20	16.77	14.16	9.98	9.73	9.11
20	0.50	38.72	21.83	13.82	13.66	12.18	9.83	7.44	7.18	6.58
	1.00	38.68	21.83	13.82	13.66	12.18	9.83	7.44	7.18	6.58
	2.00	38.60	21.83	13.82	13.65	12.17	9.83	7.43	7.18	6.58
5	0.50	6.90	2.89	1.67	2.06	1.58	1.16	1.07	0.99	0.84
	1.00	6.89	2.89	1.67	2.06	1.58	1.16	1.07	0.99	0.84
	2.00	6.88	2.89	1.67	2.06	1.58	1.16	1.07	0.99	0.84
		**CFCF**								
100	0.50	6.11	2.30	1.39	3.24	1.69	1.14	2.09	1.32	0.96
	1.00	6.11	2.30	1.39	3.24	1.69	1.14	2.09	1.32	0.96
	2.00	6.11	2.30	1.39	3.24	1.69	1.14	2.09	1.32	0.96
20	0.50	5.60	2.11	1.28	2.98	1.55	1.05	1.91	1.21	0.88
	1.00	5.60	2.11	1.28	2.98	1.55	1.05	1.91	1.21	0.88
	2.00	5.59	2.11	1.28	2.98	1.55	1.05	1.91	1.21	0.88
5	0.50	3.46	1.42	0.86	2.00	1.05	0.70	1.08	0.80	0.58
	1.00	3.46	1.42	0.86	2.00	1.05	0.70	1.08	0.80	0.58
	2.00	3.46	1.42	0.86	2.00	1.05	0.70	1.08	0.80	0.58
		**SSSS**								
100	0.50	23.61	20.38	15.85	11.80	11.30	10.19	7.87	7.71	7.31
	1.00	23.56	20.35	15.83	11.79	11.29	10.18	7.86	7.71	7.31
	2.00	23.47	20.29	15.80	11.77	11.27	10.16	7.85	7.70	7.30
20	0.50	21.15	17.35	12.82	10.58	9.95	8.67	7.05	6.85	6.37
	1.00	21.11	17.33	12.81	10.57	9.94	8.66	7.05	6.85	6.36
	2.00	21.03	17.28	12.79	10.55	9.93	8.65	7.04	6.84	6.36
5	0.50	7.75	2.95	1.71	2.09	1.62	1.20	1.08	1.01	0.85
	1.00	7.75	2.95	1.71	2.09	1.62	1.20	1.08	1.01	0.85
	2.00	7.75	2.95	1.71	2.09	1.62	1.20	1.08	1.01	0.85

**Table 8:** Non-dimensional buckling load $\bar{\lambda }=(\lambda b^{2}/E_{2}h^{3})$ for a bi-axial buckling of a composite plate having lamination scheme C and additional mass.

*a*/*h*	Concentrated Mass	CCCC								
		N_x_/N_y_ = 0			N_x_/N_y_ = 1			N_x_/N_y_ = 2		
		N_xy_ = 0	N_xy_ = 1	N_xy_ = 2	N_xy_ = 0	N_xy_ = 1	N_xy_ = 2	N_xy_ = 0	N_xy_ = 1	N_xy_ = 2
100	0.50	81.70	64.48	45.08	42.67	39.97	32.78	28.17	27.74	24.88
	1.00	81.51	64.39	45.04	42.61	39.93	32.76	28.15	27.72	24.86
	2.00	81.13	64.22	44.97	42.50	39.85	32.71	28.10	27.67	24.83
20	0.50	54.53	38.47	25.50	30.29	26.00	19.75	19.72	18.56	16.56
	1.00	54.42	38.43	25.48	30.25	25.98	19.74	19.71	18.55	16.54
	2.00	54.22	38.36	25.45	30.17	25.93	19.72	19.67	18.53	16.49
5	0.50	4.67	4.73	2.78	3.59	3.90	2.64	1.94	2.15	2.10
	1.00	4.67	4.73	2.78	3.59	3.90	2.64	1.94	2.15	2.10
	2.00	4.67	4.73	2.78	3.59	3.90	2.64	1.94	2.15	2.10
		**CCSS**								
100	0.50	58.94	50.58	37.48	33.32	32.03	27.40	23.08	22.87	21.02
	1.00	58.82	50.51	37.45	33.29	32.00	27.38	23.06	22.85	21.00
	2.00	58.58	50.36	37.38	33.21	31.93	27.34	23.02	22.82	20.98
20	0.50	43.73	33.51	23.11	24.72	22.46	17.83	16.99	16.37	14.24
	1.00	43.64	33.48	23.10	24.69	22.44	17.82	16.97	16.35	14.23
	2.00	43.47	33.41	23.07	24.64	22.40	17.80	16.95	16.33	14.21
5	0.50	4.72	4.72	2.78	3.57	3.82	2.62	1.93	2.14	2.10
	1.00	4.72	4.72	2.78	3.57	3.82	2.62	1.93	2.14	2.10
	2.00	4.72	4.72	2.78	3.57	3.81	2.62	1.93	2.14	2.10
		**SSCC**								
100	0.50	66.86	54.06	38.13	29.94	29.01	25.12	19.16	19.15	17.95
	1.00	66.71	53.98	38.09	29.90	28.98	25.11	19.15	19.14	17.94
	2.00	66.39	53.81	38.03	29.84	28.92	25.07	19.12	19.11	17.92
20	0.50	44.45	35.59	23.63	22.96	21.11	17.04	14.75	14.37	12.79
	1.00	44.45	35.57	23.61	22.93	21.10	17.03	14.73	14.36	12.79
	2.00	44.45	35.52	23.58	22.88	21.06	17.01	14.71	14.34	12.78
5	0.50	4.66	4.73	2.77	3.59	3.89	2.61	1.94	2.15	2.10
	1.00	4.66	4.73	2.77	3.59	3.89	2.61	1.94	2.15	2.10
	2.00	4.66	4.73	2.77	3.59	3.89	2.61	1.94	2.15	2.10

**Table 9:** (**a**) Non-dimensional buckling load $\bar{\lambda }=(\lambda b^{2}/E_{2}h^{3})$ for a bi-axial buckling of a composite plate having lamination scheme A and central square cutout; (**b**) Non-dimensional buckling load $\bar{\lambda }=(\lambda b^{2}/E_{2}h^{3})$ for a bi-axial buckling of a composite plate having lamination scheme A and central square cutout.

**(a)**										
***a/h***	**Cutout Size**	**CCCC**								
		**N_x_/N_y_ = 0**			**N_x_/N_y_ = 1**			**N_x_/N_y_ = 2**		
		**N_xy_ = 0**	**N_xy_ = 1**	**N_xy_ = 2**	**N_xy_ = 0**	**N_xy_ = 1**	**N_xy_ = 2**	**N_xy_ = 0**	**N_xy_ = 1**	**N_xy_ = 2**
100	0.2a	58.93	40.28	26.28	21.88	19.74	16.27	12.27	11.85	10.89
	0.4a	64.98	51.36	34.48	24.11	22.27	18.92	13.24	12.91	12.09
	0.6a	125.86	86.64	51.58	22.14	21.43	19.74	11.64	11.53	11.24
20	0.2a	40.09	23.26	14.52	15.94	13.26	10.12	8.90	8.35	7.27
	0.4a	43.29	28.69	17.52	15.36	13.48	10.77	8.32	7.98	7.23
	0.6a	61.50	41.47	24.87	14.48	13.70	12.08	7.59	7.47	7.16
5	0.2a	6.44	2.75	1.58	1.62	1.36	1.04	0.82	0.80	0.71
	0.4a	6.33	2.73	1.58	1.62	1.36	1.03	0.82	0.80	0.71
	0.6a	6.32	2.85	1.65	1.64	1.39	1.06	0.83	0.82	0.73
		**CCSS**								
100	0.2a	57.76	40.11	25.82	19.57	17.91	15.02	10.66	10.37	9.67
	0.4a	62.86	49.61	32.81	23.37	21.59	18.34	12.34	12.07	11.39
	0.6a	103.22	58.35	35.75	17.81	17.06	15.40	9.62	9.49	9.15
20	0.2a	38.21	22.93	14.30	14.61	12.47	9.68	7.86	7.49	6.69
	0.4a	40.98	26.50	16.16	15.38	13.38	10.41	8.26	8.03	7.15
	0.6a	56.28	30.15	18.26	9.65	9.20	8.23	5.20	5.13	4.93
5	0.2a	6.53	2.75	1.58	1.61	1.36	1.04	0.81	0.79	0.70
	0.4a	6.46	2.73	1.57	1.61	1.35	1.02	0.81	0.79	0.70
	0.6a	6.33	2.76	1.59	1.61	1.35	1.02	0.81	0.80	0.70
		**SSCC**								
100	0.2a	19.44	15.97	11.83	8.09	7.72	6.92	5.01	4.92	4.68
	0.4a	11.07	10.38	8.99	5.39	5.30	5.05	3.52	3.50	3.42
	0.6a	6.42	6.39	6.30	4.73	4.72	4.67	3.66	3.65	3.63
20	0.2a	16.85	12.85	9.00	7.07	6.59	5.66	4.37	4.25	3.94
	0.4a	9.63	8.75	7.21	4.87	4.73	4.39	3.20	3.15	3.04
	0.6a	5.33	5.24	5.02	3.93	3.90	3.83	3.12	3.11	3.06
5	0.2a	6.42	2.74	1.57	1.62	1.36	1.04	0.82	0.80	0.70
	0.4a	4.22	2.73	1.57	1.62	1.36	1.03	0.82	0.80	0.71
	0.6a	2.11	1.94	1.60	1.61	1.39	1.06	0.83	0.81	0.73
(**b**)										
***a/h***	**Cutout Size**	**CCFF**								
		**N_x_/N_y_ = 0**			**N_x_/N_y_ = 1**			**N_x_/N_y_ = 2**		
		**N_xy_ = 0**	**N_xy_ = 1**	**N_xy_ = 2**	**N_xy_ = 0**	**N_xy_ = 1**	**N_xy_ = 2**	**N_xy_ = 0**	**N_xy_ = 1**	**N_xy_ = 2**
100	0.2a	57.52	35.18	21.57	13.23	12.66	11.41	6.88	6.79	6.57
	0.4a	62.79	37.52	22.06	11.62	11.06	9.93	6.09	6.00	5.79
	0.6a	81.21	36.95	21.32	6.95	6.83	6.49	3.56	3.55	3.49
20	0.2a	38.18	19.14	11.41	8.72	8.10	7.02	4.58	4.49	4.26
	0.4a	40.92	19.73	11.44	6.95	6.62	5.70	3.70	3.64	3.47
	0.6a	47.69	18.40	10.46	3.79	3.70	3.49	1.94	1.93	1.90
5	0.2a	6.40	2.70	1.55	1.61	1.33	1.01	0.81	0.79	0.70
	0.4a	6.46	2.68	1.53	1.55	1.33	1.00	0.81	0.78	0.69
	0.6a	6.51	2.54	1.48	0.90	0.85	0.74	0.47	0.46	0.44
**CFCF**										
100	0.2a	6.07	2.17	1.28	2.57	1.44	0.98	1.39	1.01	0.77
	0.4a	4.77	1.82	1.08	2.27	1.25	0.85	1.22	0.89	0.67
	0.6a	3.05	1.36	0.82	1.75	0.96	0.65	0.95	0.69	0.53
20	0.2a	5.24	1.86	1.09	2.23	1.24	0.84	1.21	0.88	0.67
	0.4a	4.01	1.55	0.92	1.94	1.06	0.72	1.05	0.76	0.58
	0.6a	2.48	1.17	0.71	1.46	0.82	0.56	0.78	0.58	0.45
5	0.2a	3.09	1.09	0.63	1.20	0.70	0.48	0.64	0.48	0.37
	0.4a	2.13	0.89	0.53	0.97	0.58	0.40	0.52	0.40	0.31
	0.6a	1.13	0.65	0.40	0.67	0.44	0.31	0.35	0.29	0.23
**SSSS**										
100	0.2a	15.29	13.43	10.57	7.40	7.12	6.47	4.81	4.72	4.50
	0.4a	7.64	7.31	6.58	3.97	3.92	3.78	2.67	2.65	2.61
	0.6a	3.94	3.92	3.85	2.34	2.33	2.31	1.60	1.60	1.59
20	0.2a	12.92	10.76	8.03	6.30	5.95	5.22	4.10	4.00	3.73
	0.4a	6.59	6.18	5.35	3.49	3.42	3.24	2.35	2.32	2.26
	0.6a	3.39	3.35	3.25	2.09	2.08	2.05	1.43	1.42	1.41
5	0.2a	5.22	2.73	1.57	1.61	1.36	1.03	0.81	0.79	0.71
	0.4a	3.21	2.55	1.57	1.62	1.35	1.02	0.81	0.79	0.70
	0.6a	1.83	1.72	1.50	1.16	1.12	1.01	0.72	0.71	0.67

**Table 10:** Non-dimensional buckling load $\bar{\lambda }=(\lambda b^{2}/E_{2}h^{3})$ for a bi-axial buckling of a composite plate having lamination scheme B and central square cutout.

*a*/*h*	Cutout Size	CCCC								
		N_x_/N_y_ = 0			N_x_/N_y_ = 1			N_x_/N_y_ = 2		
		N_xy_ = 0	N_xy_ = 1	N_xy_ = 2	N_xy_ = 0	N_xy_ = 1	N_xy_ = 2	N_xy_ = 0	N_xy_ = 1	N_xy_ = 2
100	0.2a	59.92	43.18	29.59	30.27	26.69	21.44	19.74	18.59	16.31
	0.4a	62.70	52.24	38.94	33.16	30.81	26.40	21.19	20.52	18.93
	0.6a	97.67	93.52	81.87	52.71	51.02	46.97	28.25	27.98	27.25
20	0.2a	43.47	28.14	18.38	22.90	18.64	14.02	14.56	13.19	10.99
	0.4a	43.98	33.21	23.00	24.96	21.49	16.85	15.03	14.11	12.31
	0.6a	56.77	48.97	33.53	34.24	30.64	24.45	18.14	17.55	16.15
5	0.2a	6.63	2.92	1.71	2.09	1.63	1.18	1.08	1.01	0.86
	0.4a	6.55	2.91	1.71	2.08	1.61	1.18	1.08	1.01	0.85
	0.6a	6.57	3.05	1.78	2.08	1.64	1.22	1.09	1.02	0.87
		**CCSS**								
100	0.2a	46.37	36.16	25.84	26.38	23.64	19.28	18.18	17.14	15.04
	0.4a	47.18	39.91	30.18	24.63	23.13	20.12	16.12	15.65	14.52
	0.6a	79.92	63.48	44.81	16.34	16.09	15.43	9.00	8.95	8.83
20	0.2a	34.12	24.20	16.40	19.42	16.43	12.67	13.19	12.04	10.10
	0.4a	34.92	26.85	18.75	17.23	15.56	12.81	10.73	10.27	9.24
	0.6a	50.06	34.61	22.62	9.81	9.55	8.92	5.35	5.31	5.19
5	0.2a	6.62	2.93	1.70	2.09	1.62	1.19	1.07	1.01	0.85
	0.4a	6.59	2.91	1.70	2.08	1.60	1.18	1.07	1.00	0.84
	0.6a	6.56	2.95	1.72	1.85	1.61	1.17	1.02	0.97	0.85
		**SSCC**								
100	0.2a	39.63	29.62	20.69	17.25	15.95	13.56	10.95	10.58	9.73
	0.4a	29.06	25.61	20.38	14.74	14.18	12.89	9.71	9.54	9.10
	0.6a	14.69	14.52	14.03	11.67	11.58	11.32	9.60	9.55	9.41
20	0.2a	30.94	21.57	14.52	13.95	12.46	10.14	8.85	8.42	7.51
	0.4a	21.00	17.94	13.81	11.70	11.02	9.64	7.86	7.64	7.10
	0.6a	9.21	9.00	8.47	7.50	7.38	7.07	6.30	6.23	6.03
5	0.2a	6.68	2.93	1.70	2.08	1.63	1.18	1.08	1.01	0.86
	0.4a	4.81	2.90	1.70	2.08	1.61	1.19	1.08	1.01	0.85
	0.6a	2.27	2.08	1.71	1.76	1.63	1.21	1.09	1.02	0.87

**Table 11:** Non-dimensional buckling load $\bar{\lambda }=(\lambda b^{2}/E_{2}h^{3})$ for a bi-axial buckling of a composite plate having lamination scheme C and central square cutout.

*a*/*h*	Cutout Size	CCCC								
		N_x_/N_y_ = 0			N_x_/N_y_ = 1			N_x_/N_y_ = 2		
		N_xy_ = 0	N_xy_ = 1	N_xy_ = 2	N_xy_ = 0	N_xy_ = 1	N_xy_ = 2	N_xy_ = 0	N_xy_ = 1	N_xy_ = 2
100	0.2a	57.59	47.63	34.31	29.99	28.67	24.24	19.75	19.62	18.02
	0.4a	57.41	52.03	40.91	30.92	30.31	27.24	20.19	20.21	19.29
	0.6a	76.20	75.01	69.17	52.70	52.80	49.61	29.97	30.11	29.74
20	0.2a	40.97	30.71	20.93	22.55	20.13	15.80	14.65	14.08	12.21
	0.4a	38.19	32.06	23.58	22.79	21.13	17.46	14.40	14.09	12.80
	0.6a	44.23	40.66	33.37	33.77	31.74	27.26	18.03	17.82	16.84
5	0.2a	4.63	4.71	2.63	3.48	3.79	2.59	1.92	2.14	2.09
	0.4a	4.60	4.68	2.56	3.39	3.85	2.70	1.88	2.14	2.07
	0.6a	4.63	4.71	2.28	3.38	3.88	2.83	1.89	2.14	2.09
		**CCSS**								
100	0.2a	43.52	38.14	28.80	24.81	24.00	20.76	17.14	17.05	15.77
	0.4a	42.38	39.29	31.78	22.96	22.72	20.82	15.36	15.41	14.84
	0.6a	67.10	65.27	57.53	24.38	24.62	24.01	13.68	13.80	13.78
20	0.2a	33.39	26.66	18.84	19.14	17.61	14.26	13.06	12.66	11.14
	0.4a	31.48	27.13	20.36	17.23	16.50	14.21	11.12	11.02	10.26
	0.6a	42.82	39.54	31.35	15.03	14.95	13.98	8.20	8.25	8.13
5	0.2a	4.74	4.68	2.63	3.50	3.63	2.57	1.93	2.14	2.08
	0.4a	4.68	4.67	2.56	3.41	3.62	2.61	1.88	2.14	2.03
	0.6a	4.66	4.70	2.28	3.40	3.42	2.70	1.85	1.98	1.84
		**SSCC**								
100	0.2a	49.82	40.95	29.34	22.31	21.75	19.05	14.21	14.24	13.45
	0.4a	40.37	37.33	29.96	20.20	20.09	18.62	13.18	13.26	12.88
	0.6a	29.27	29.47	28.37	22.63	22.83	22.38	17.65	17.80	17.64
20	0.2a	37.49	28.07	19.14	17.65	16.46	13.53	11.27	11.07	10.01
	0.4a	28.18	24.76	18.89	15.66	15.12	13.23	10.29	10.22	9.59
	0.6a	17.87	17.62	16.16	14.81	14.73	13.82	12.20	12.23	11.81
5	0.2a	4.62	4.72	2.63	3.48	3.80	2.55	1.92	2.14	2.08
	0.4a	4.60	4.53	2.56	3.39	3.64	2.64	1.88	2.14	2.07
	0.6a	4.28	4.05	2.28	3.46	3.47	2.76	1.90	2.13	2.08
